# Low expression of CADPS predicts poor prognosis in pediatric acute lymphoblastic leukemia without fusion genes

**DOI:** 10.17305/bb.2025.12254

**Published:** 2025-05-30

**Authors:** Bin Zhang, Chaoran Shi, Xiuxiu Wang, Jiajia Mi, Runan Wang, Shuang Li, Jiawei Yang, Qiuying He, Yujiao Wang, Zuofei Chi, Liangchun Hao

**Affiliations:** 1 Department of Pediatrics, Shengjing Hospital of China Medical University, Shenyang, Liaoning, China; 2 Department of Translational Medicine Center, Chigene (Beijing) Translational Medical Research Center Co., Beijing, China

**Keywords:** Pediatric acute lymphoblastic leukemia, ALL, fusion gene negative, FG-negative, next-generation sequencing, prognostic biomarker

## Abstract

Pediatric acute lymphoblastic leukemia (ALL) is among the most prevalent hematological malignancies in children. Despite an overall cure rate approaching 90%, a subset of patients still experiences relapse, even with advanced therapeutic interventions. Research into the molecular characteristics and prognostic markers of fusion gene-negative (FG-negative) pediatric ALL remains limited. To address this gap, we performed whole-exome sequencing (WES) and whole-transcriptome sequencing (RNA-seq) on 54 FG-negative ALL cases from our center. Our results indicated that neither specific mutations nor tumor mutational burden significantly influenced relapse risk. Notably, we identified a significant downregulation of CADPS in FG-negative pediatric ALL patients who relapsed. The expression levels and prognostic significance of CADPS were further validated using data from the Therapeutically Applicable Research to Generate Effective Treatments (TARGET) cohort, where lower CADPS expression was associated with reduced event-free survival (EFS) and overall survival (OS) (*P* < 0.001 for both). Cox regression analyses were subsequently employed to identify OS-related factors and to construct a prognostic prediction model. Notably, this model demonstrated a significant correlation with therapeutic targets. In conclusion, our findings support the potential of CADPS expression as a novel biomarker for prognostic stratification in FG-negative pediatric ALL patients.

## Introduction

Fusion genes (FGs) in acute leukemia—and in all known leukemias—are considered initiating variants and critical factors in tumorigenesis [1]. These genes are stable in tumor cells and have been widely used as molecular markers for the diagnosis, classification, risk stratification, and targeted therapy of leukemia [2]. Given the pivotal role of FGs in the onset and progression of leukemia, the latest edition of the World Health Organization (WHO) classification of hematopoietic and lymphoid tissue tumors includes dozens of fusion genes as essential molecular typing features [3]. Current clinical trial data suggest that the cure rate for acute lymphoblastic leukemia (ALL) has exceeded 90% [4]. However, further improvements in prognosis will depend on the expanded use of molecular, immune, and cellular therapies, as well as more precise risk stratification. Recognizing the important role of FGs in the etiology and progression of ALL, the most recent version of the National Comprehensive Cancer Network (NCCN) guidelines for Pediatric ALL incorporates several FGs as genetic risk factors. These are grouped according to risk after diagnosis and should be assessed by considering factors such as patient age, peripheral white blood cell count, extramedullary leukemia involvement, immunophenotype, tumor cytogenetics, and treatment response at diagnosis [5]. High-risk FGs, such as *MLL-AF4* or other MLL rearrangements, *MEF2D* rearrangements, and *TCF3-HLF* fusions, necessitate treatment regimens of varying intensity based on clinical risk. While a range of FGs influencing pediatric ALL prognosis have been identified, only approximately 60% of childhood ALL cases show fusion gene positivity [6, 7]. Next-generation sequencing (NGS) has become a vital tool for exploring the molecular pathogenesis of hematological malignancies and guiding clinical decision-making, owing to its high throughput, sensitivity, and capacity for ongoing monitoring. When combined with whole-exome sequencing (WES) and RNA-seq, a single assay can simultaneously detect gene fusions, mutations, and expression profiles. In this study, we employed these technologies to investigate adverse prognostic factors in children with FG-negative ALL treated at our center. We validated our findings using clinical and sequencing data from pediatric ALL patients in the TARGET database, with the goal of establishing a risk stratification model for FG-negative cases and improving diagnostic and therapeutic precision in clinical practice.

## Materials and methods

### Patients

From May 2021 to December 2023, a retrospective cohort of 120 childhood ALL cases was enrolled in this study. Diagnosis and treatment were conducted in accordance with the Chinese Children’s Leukemia Group protocol—China Acute Lymphoblastic Leukemia 2018 (CCLG-ALL2018). Of the 120 patients identified, 66 with fusion genes detected by RNA sequencing were excluded. The remaining 54 cases were categorized into two groups: complete response (CR) and relapse. Relapse was defined as the presence of > 5% blast cells in peripheral blood or bone marrow, or involvement of any extramedullary site, following a CR. Clinical data, including gender, karyotype, and age at diagnosis, were collected. The median follow-up period was 1.61 years (range: 0.25–2.78 years). This study was approved by the Medical Ethics Committee of Shengjing Hospital, China Medical University, in accordance with the Declaration of Helsinki (Approval number: 2024PS1659K).

### Sample preparation

Bone marrow samples were collected in EDTA tubes, and half of the aspirate was transferred to tubes containing RNA stabilizing solution (Tiangen, China). Matched nail and bone marrow samples were processed for DNA extraction within 72 h using the Blood Genome Column Medium Extraction Kit (Cowin Biotech, China). All DNA samples (from bone marrow and nails) underwent quality assessment using the Qubit dsDNA HS Assay Kit (Invitrogen) on a Qubit 2.0 fluorometer, followed by 0.8% agarose gel electrophoresis. DNA samples were considered acceptable if they met both of the following criteria: a final concentration > 10 ng/µL and a total quantity > 1200 ng. RNA was extracted from bone marrow using the Blood RNAprep Pure Kit (Tiangen). Total RNA was required to meet two quality thresholds: a minimum yield of 2 µg and a RNA Integrity Number (RIN) ≥ 7.

### Sequencing data acquisition

Whole-exome capture for somatic sequencing was performed using the xGen Exome Research Panel v1.0 (IDT, Iowa, USA). Captured libraries were enriched through ten cycles of PCR using PreLM PCR Oligos (Kapa Biosystems, Inc.). Library quality and concentration were assessed with the Qubit dsDNA HS Detection Kit (Invitrogen, Carlsbad, CA, USA) on a Qubit 2.0 Fluorometer. For transcriptome sequencing, approximately 500 ng of RNA was used to prepare libraries with the mRNA-seq Library Preparation Kit for Illumina (ABclonal, Woburn, MA, USA). Final libraries were also evaluated using the Qubit dsDNA HS Assay Kit on a Qubit 2.0 Fluorometer. Both whole-exome and whole-transcriptome sequencing were conducted on the DNBSEQ-T7 platform (BGI, Shenzhen, China), facilitated by the Beijing Chigene Translational Medicine Research Center Co., Ltd.

### Genome alignment and variants calling

Qualified clean data were obtained following the meticulous removal of low-quality sequencing reads and adapter sequences. The resulting clean reads were aligned to the human reference genome (GRCh37/hg19) in FASTQ format using the Burrows-Wheeler Aligner (BWA-MEM). Somatic variant calling was performed with Mutect2 in GATK4, which leverages local assembly, realignment, and Bayesian statistics to accurately identify single nucleotide variants (SNVs) and insertions/deletions (indels). SNPs and indels were further filtered according to stringent in-house quality criteria to ensure the reliability of the detected variants. The custom bioinformatics pipeline integrated the manufacturer’s analytical framework with an internally developed variant annotation algorithm, which utilized both a proprietary database (Chigene) and public repositories (including 1000 Genomes, gnomAD, ClinVar, HGMD, and TCGA) to identify core variants. Pathogenicity of missense mutations was evaluated using a range of prediction tools, including SIFT, PolyPhen, LRT, MutationTaster, FATHMM, PROVEAN, REVEL, and CADD.

### RNA-seq analysis

Clean RNA-seq datasets were obtained using processing methodologies consistent with those established for WES. Fusion variants were identified using STAR-Fusion [8] (v1.10.1) and Arriba [9] (v2.4.0) with default parameters, based on alignments generated by STAR. Differentially expressed genes (DEGs) were detected using the DESeq2 package, applying a threshold of log_2_|fold change| > 1 and a false discovery rate–adjusted *P* value (padj) of less than 0.05.

### Real-time PCR (RT-PCR)

For gene fusion validation, leukemia-associated fusion genes were analyzed using a Leukemia-Associated Fusion Gene Detection Kit (fluorescence-based RT-PCR method; Yuanqi Bio-Pharmaceutical Co. Ltd., Shanghai, China) according to the manufacturer’s protocol. PCR amplification was performed on an ABI 7500 Real-Time PCR System (Applied Biosystems). A complete list of the genes examined by RT-PCR is provided in Table S1.

### Public data download and data preprocessing

Validation cohort data for pediatric ALL bone marrow samples were retrieved from the cBioPortal database (https://www.cbioportal.org/), which provides publicly available data. A total of 203 RNA-seq samples from the ALL Phase II cohort of the TARGET study, along with corresponding clinical data, were downloaded. The clinical information included age, sex, minimal residual disease (MRD) status, fusion gene status, EFS time and first outcome event, overall survival (OS), and survival status. During subsequent data preprocessing, peripheral blood samples and other inappropriate cases were excluded.

### Gene Set Enrichment Analysis (GSEA)

GSEA is a computational method used to determine whether a predefined set of genes shows statistically significant and consistent differences between two biological states. In this study, the GSEA database (www.broadinstitute.org/gsea) was used to analyze differentially expressed genes between low- and high-risk pediatric ALL patients, thereby identifying enriched signaling pathways associated with disease risk. A *P* value of less than 0.05 was considered statistically significant.

### Prediction of drug sensitivity

The pRRophetic package was used to predict the sensitivity to chemotherapy and targeted drugs for comparing high- and low-risk ALL groups, based on data from the GDSC database (https://www.cancerrxgene.org/) [10].

### Ethical statement

The ethical protocol for this study was approved by the Ethics Committee of Shengjing Hospital, China Medical University (Approval Number: 2024PS1659K). Written informed consent for participation was obtained from the legal guardians or next of kin of the participants. Additionally, explicit written consent was obtained from the guardians for the publication of any potentially identifiable images or data included in this article.

### Statistical analysis

Statistical analyses and graphical visualizations were performed using R (version 4.1.3), incorporating the maftools, survival, timeROC, and ggplot2 packages, as well as GraphPad Prism (version 10.1.2). Statistical significance was assessed using the Mann-Whitney *U* test, Yates’ continuity-corrected chi-square test, Fisher’s exact test, and the Log-Rank test. A *P* value of < 0.05 was considered statistically significant.

## Results

### Patient cohort and the overview

**Figure 1.**
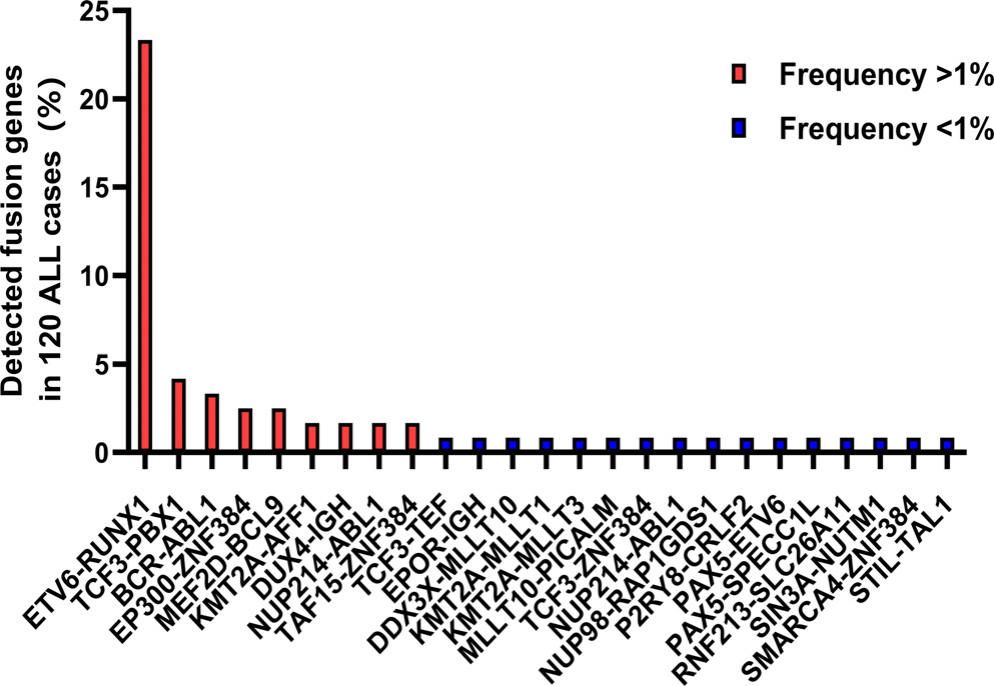
**The distribution of detected fusion genes in 120 cases ALL cohort.** Fusion genes with frequency greater than one percent from high to low are *ETV6-RUNX1, TCF3-PBX1, BCR-ABL1, EP300-ZNF384, MEF2D-BCL9, KMT2A-AFF1, DUX4-IGH, NUP214-ABL1* and *TAF15-ZNF384*. ALL: Acute lymphoblastic leukemia.

For the 120 pediatric ALL bone marrow samples, the mean WES depth was 1,095× before deduplication and 929× after deduplication. Matched nail samples showed a mean WES depth of 197× pre-deduplication and 188× post-deduplication. RNA-seq data from the bone marrow samples yielded an average of 65.34 million reads before deduplication and 60.01 million reads after, indicating high sequencing quality across all datasets. NGS analysis identified somatic mutations clearly associated with ALL, gene fusions, or pathogenic/likely pathogenic germline mutations in 105 of the 120 cases, yielding an overall positivity rate of 87.5%. Gene fusion variants were detected in 66 patients, with the distribution of fusion types shown in Figure 1. The five most frequent fusions were *ETV6-RUNX1* (23.33%), *TCF3-PBX1* (4.17%), *BCR-ABL1* (3.33%), KMT2A/MLL rearrangements (3.33%), *EP300-ZNF384* (2.5%), and *MEF2D-BCL9* (2.5%). Previous studies suggest that the *EP300-ZNF384* fusion subgroup has a better prognosis compared to other pre-ALL subtypes, consistent with findings from a Japanese cohort [11, 12]. In contrast, KMT2A/MLL rearrangements represent a high-risk subtype of pediatric ALL, commonly involving chaperone genes such as AFF1, MLLT1, MLLT3, and MLLT10 [13]. The *MEF2D-BCL9* fusion, although rare, is associated with poor prognosis and may represent a potential target for novel molecular therapies [14].

### Genomic and transcriptome characteristics of fusion-negative ALL

**Figure 2.**
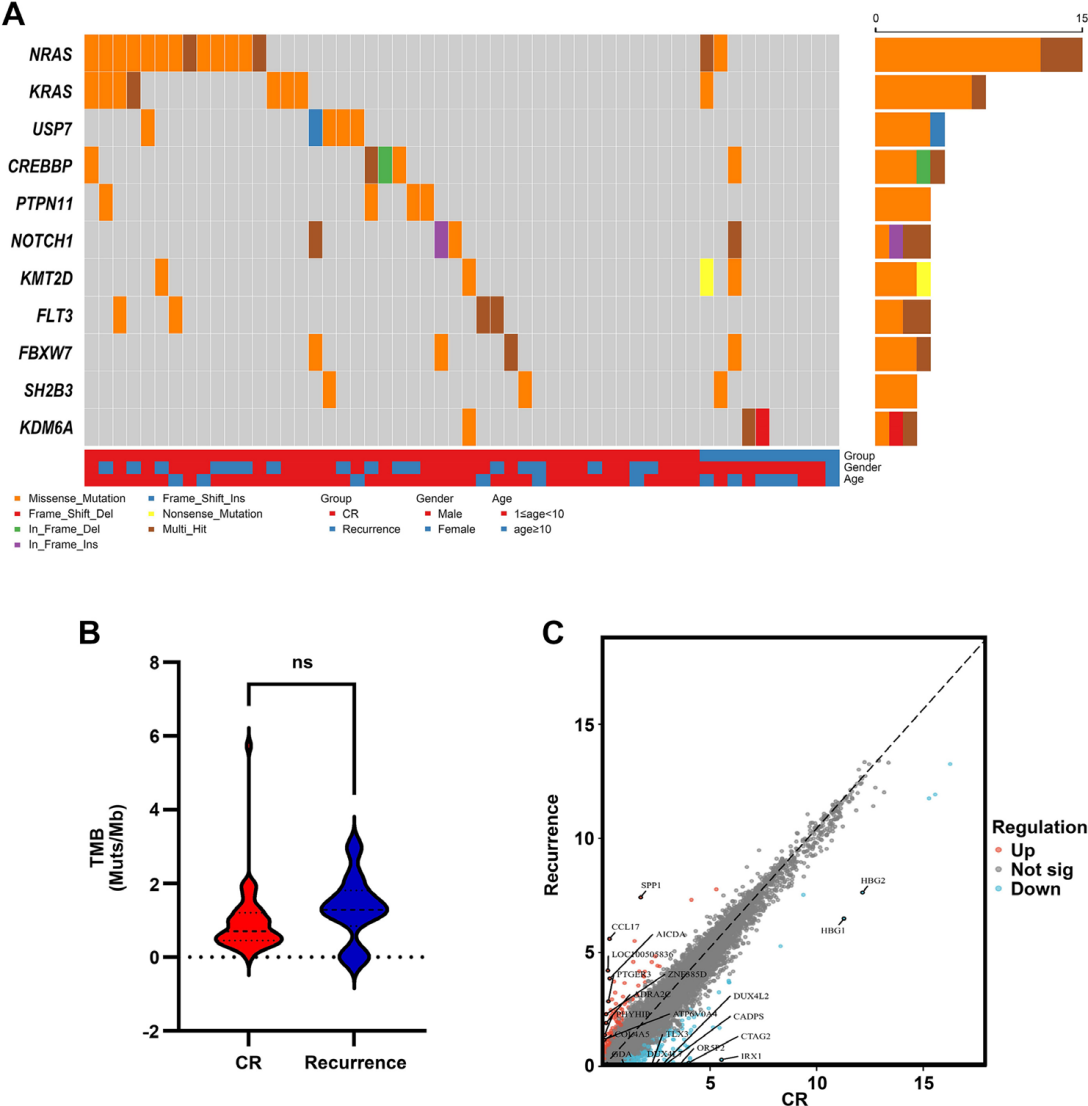
**Genomic and transcriptomic characteristics of the FG-negative ALL cohort in our institution.** (A) A landscape map of mutation was displayed in the top 10 mutation in 54 fusion gene negative ALL; (B) Violin plot of tumor mutation burden difference in the CR group and Recurrence group (Mann-Whitney *U* test); (C) Volcano plot shows the differential expressed genes between the CR and recurrence groups. The grey dots represent genes without different expression between two groups (Not sig), the red dots represent differentially up-regulated genes (Up), and the blue dots represent differentially down-regulated genes (Down) in recurrence group.

Among the 120 primary treatment-naïve ALL cases at our center, 54 (45%) were detected without any FGs. Of these 54 cases, 44 achieved complete remission (CR), while 10 experienced relapse during follow-up. Detailed characteristics of the included patients are summarized in Table 1. WES results for these 54 children (Table S2) revealed that common mutations—including *NRAS*, *KRAS*, and *USP7*, among others—ranked among the top 10 mutations by descending frequency. This analysis indicated that the differences in mutation frequencies between the CR and relapse groups were not statistically significant (Figure 2A, Table 2). *NRAS* and *KRAS* exhibited the highest mutation rates in our cohort, consistent with findings from other pediatric cohorts in China [15]. Additionally, statistical analysis showed no significant difference in tumor mutational burden (TMB) between the two groups (Figure 2B). We then compared gene expression profiles between the CR and relapse groups. Using DESeq software, we found that 35 genes were significantly upregulated and 317 genes were significantly downregulated in the relapse group (Figure 2C).

**Table 1:** Clinical information of patients with fusion negative ALL

**Characteristics**	**CR group**	**Recurrence group**	***P* value**
Age (years)			0.0057
1≤age<10	38	4	
age≥10	6	6	
Sex			0.1731
Male	27	9	
Female	17	1	
Karyotype			0.2499
Normal karyotype	32	8	
Hyperdiploid	9	1	
Hypodiploid	0	1	
N/A	3	0	
Cell of origin			0.4878
B cell	41	8	
T cell	3	2	
WBC counts (x 10^9^/L) (Mean ± SEM)	51.32±21.4	116.69±56.4	0.028
CNSL			0.81
Yes	1	1	
No	43	9	
Risk stratification			0.0214
Low risk	11	0	
Intermediate risk	27	5	
High risk	6	5	

*P* value was calculated using the Mann-Whitney *U* test for non-normally distributed continuous variables (WBC counts). CR: Complete response; WBC: White blood cells; SEM: Standard error of mean; CNSL: Central nervous system lymphoma.

**Table 2:** Characteristics of top 10 mutation in 54 FG-negative pediatric ALL patients of our center grouped by the outcomes

**Gene**	**Status**	**CR group**	**Recurrence group**	***P* value**
*NRAS*	Mut	13	2	0.828
	Wildtype	31	8	
*KRAS*	Mut	7	1	0.9854
	Wildtype	37	9	
*USP7*	Mut	5	0	0.6067
	Wildtype	39	10	
*CREBBP*	Mut	4	1	0.6067
	Wildtype	40	9	
*PTPN11*	Mut	4	0	0.322
	Wildtype	40	10	
*NOTCH1*	Mut	3	1	0.7474
	Wildtype	41	9	
*KMT2D*	Mut	2	2	0.3098
	Wildtype	42	8	
*FLT3*	Mut	4	0	0.322
	Wildtype	40	10	
*FBXW7*	Mut	3	1	0.7474
	Wildtype	41	9	
SH2B3^*^	Mut	2	1	0.9323
	Wildtype	42	9	
KDM6A^*^	Mut	1	2	0.1486
	Wildtype	43	8	

^*^SH2B3 and *KDM6A* shared same mutation frequency in FG negative pediatric ALL patients. ALL: Acute lymphoblastic leukemia; CR: Complete response; FG: Fusion gene.

### Prognostic significance of CADPS expression in pediatric ALL

**Figure 3.**
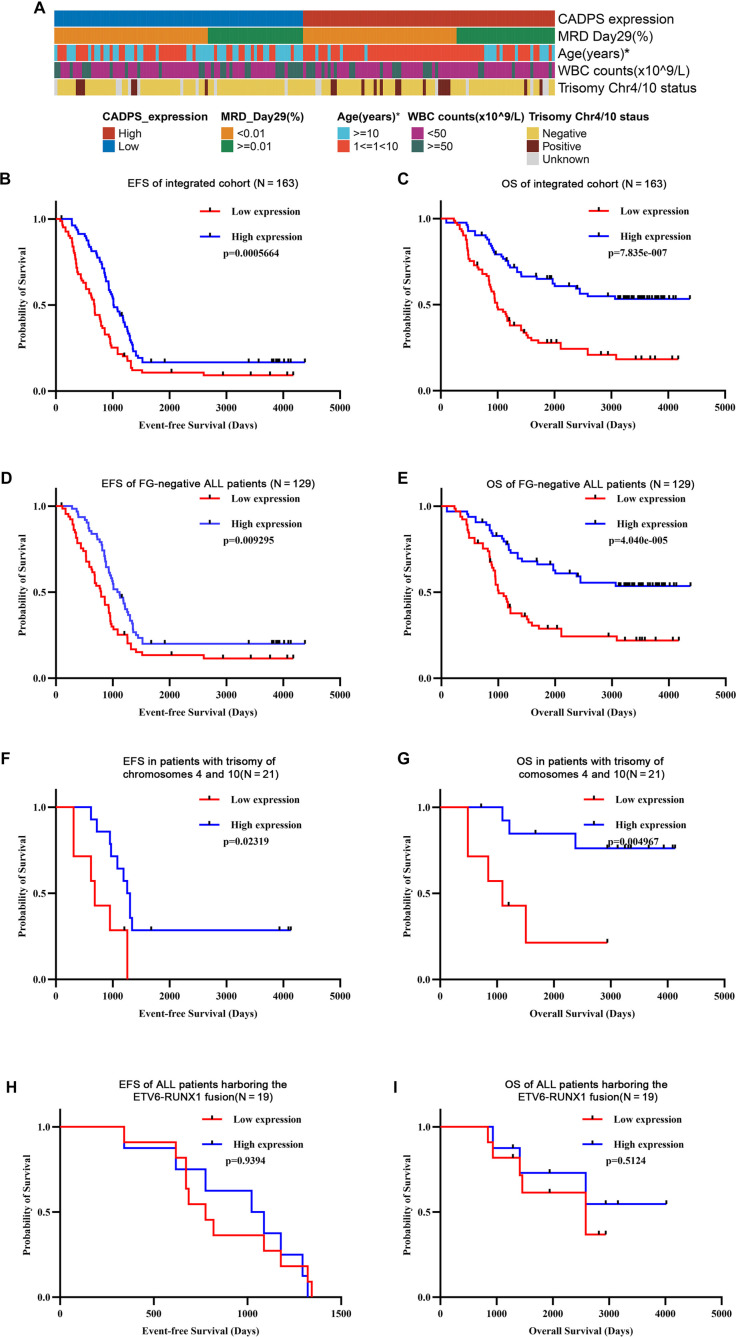
**Kaplan–Meier curves analysis of the association between EFS and OS for the low and high CADPS expression in the TARGET ALL dataset (*N* ═ 163).** (A) The correlation between CADPS expression and current classification criteria was analyzed using the chi-square test (*P < 0.05); (B) Kaplan–Meier curves for EFS of integrated cohort (high-CADPS: *N* ═ 82; low-CADPS: *N* ═ 81); (C) Kaplan–Meier curves for OS of integrated cohort (high-CADPS: *N* ═ 82; low-CADPS: *N* ═ 81); (D) Kaplan–Meier curves for EFS of FG-negative ALL patients (high-CADPS: *N* ═ 64; low-CADPS: *N* ═ 65); (E) Kaplan–Meier curves for OS of FG-negative ALL patients (high-CADPS: *N* ═ 64; low-CADPS: *N* ═ 65); (F) Kaplan–Meier curves for EFS in patients with trisomy of chromosomes 4 and 10 (high-CADPS: *N* ═ 10; low-CADPS: *N* ═ 11); (G) Kaplan–Meier curves for OS in patients with trisomy of chromosomes 4 and 10 (high-CADPS: *N* ═ 10; low-CADPS: *N* ═ 11); (H) Kaplan–Meier curves for EFS of ALL patients harboring the *ETV6-RUNX1* fusion (high-CADPS: *N* ═ 9; low-CADPS: *N* ═ 10); (I) Kaplan–Meier curves for OS of ALL patients harboring the *ETV6-RUNX1* fusion (high-CADPS: *N* ═ 9; low-CADPS: *N* ═ 10).

Among the differentially expressed genes, calcium-dependent activator protein for secretion (CADPS) emerged as a key candidate due to its marked downregulation in the recurrence group and its previously reported tumor-suppressive roles in other cancers. Specifically, CADPS downregulation has been observed in high-grade serous ovarian cancer [16], cholangiocarcinoma [17], and hepatocellular carcinoma [18], suggesting a conserved tumor-suppressive function across malignancies. We hypothesized that CADPS acts as a tumor suppressor gene (TSG) in ALL. To investigate this hypothesis, we analyzed the survival impact of CADPS expression using data from 163 cases in the TARGET ALL Phase II cohort. The cohort was divided into low and high CADPS expression groups using the median expression value as the cutoff. Differential CADPS expression showed a significant association with age stratification (*P* < 0.05), but not with MRD on Day 29 (*P* ═ 1.00), WBC counts (*P* ═ 0.46), or cytogenetic abnormalities such as trisomy 4/10 (*P* ═ 0.16) (Figure 3A). These findings suggest that low CADPS expression may define a unique molecular subtypeof ALL. Kaplan-Meier survival analysis revealed that both EFS and OS were significantly lower in the low-CADPS expression group compared to the high-expression group across the entire cohort (Figure 3B and 3C). Subgroup analyses further demonstrated that low CADPS expression predicted poorer prognosis in fusion-negative ALL patients (Figure 3D and 3E). Similar trends were observed in patients with trisomy of chromosomes 4 and 10, but not in those with the *ETV6-RUNX1* fusion (Figure 3F–3I).

### Evaluation of CADPS expression for prognostic prediction ability

**Figure 4.**
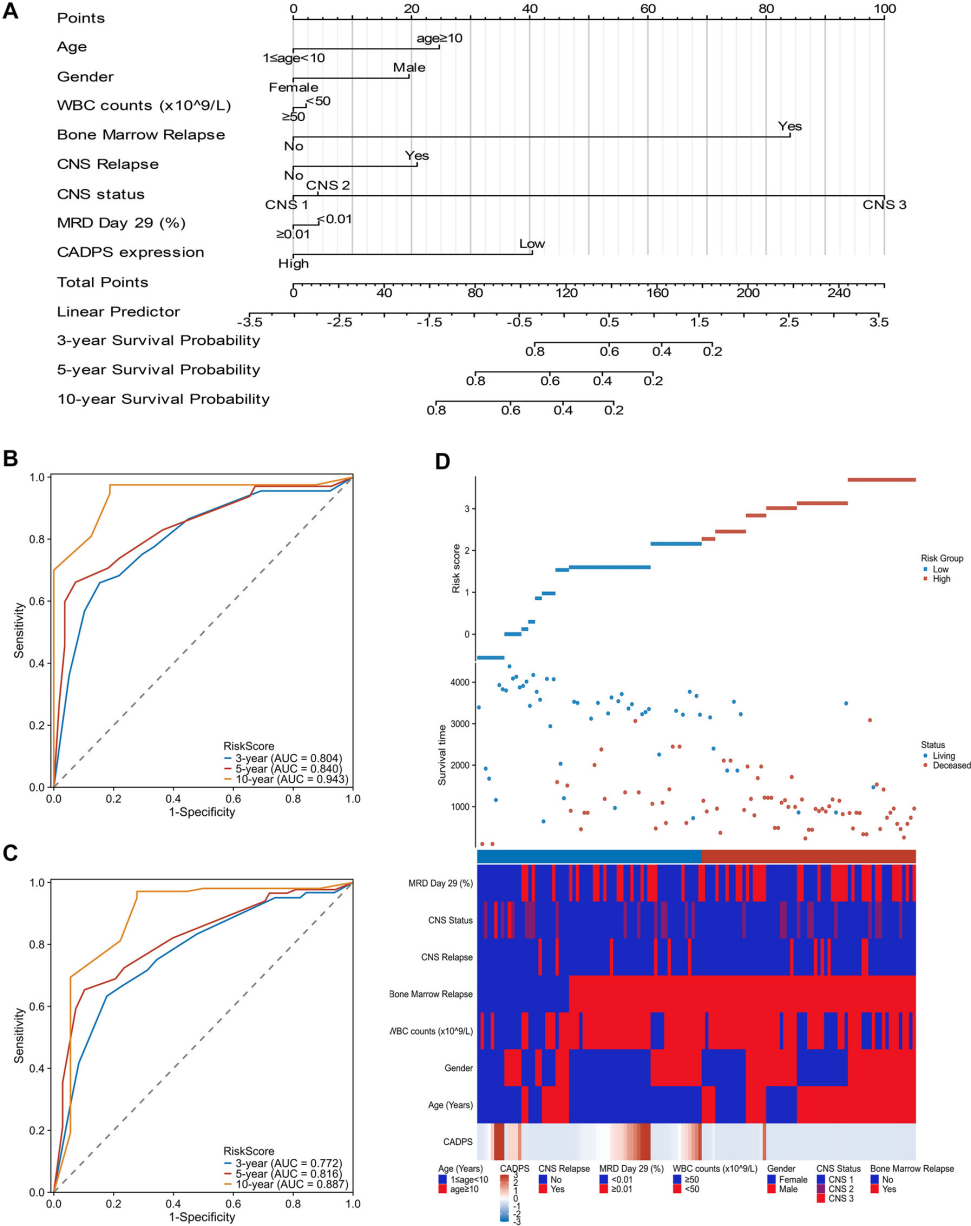
**Prognostic significance of CADPS in ALL from the TARGET ALL phase II cohort.** (A) Nomogram integrated CADPS expression and other clinical variables for prediction model; (B) Time-dependent ROCcurve analysis for OS of FG-negative ALL dataset. The area under the curve (AUC) for 3-year, 5-year, and 10-year OS was found to be 0.804, 0.840, and 0.943 for fusion-negative ALL patients, respectively; (C) Time-dependent ROC curve analysis for OS of entire ALL dataset. The corresponding AUCs for 3-year, 5-year, and 10-year OS of the entire ALL population were 0.772, 0.816, and 0.887, respectively; (D) The distribution of risk score and survival status corresponding to the expression of CADPS and other clinicopathological features in FG-negative ALL patients.

Consistent with survival analysis methodology, patients from the TARGET ALL Phase II cohort were stratified into low- and high-CADPS expression groups using the median expression level as the cutoff. We included gender, age, white blood cell (WBC) count, bone marrow relapse, central nervous system (CNS) relapse, CNS status, Day 29 MRD status, and CADPS expression in both univariate and multivariate Cox regression analyses. The results indicated that low CADPS expression was an independent prognostic predictor of OS for the entire pediatric ALL cohort (HR ═ 2.329, 95% CI: 1.493–3.632, *P* < 0.001) and for FG-negative ALL patients (HR ═ 2.350, 95% CI: 1.406–3.930, *P*< 0.001) (Tables 3–4).Using clinical variables from 129 FG-negative ALL patients, we applied the Cox method to generate a nomogram plot and calculate risk scores (Figure 4A). ROC curve analysis showed that the AUC for OS at 3, 5, and 10 years was 0.804, 0.840, and 0.943, respectively, in fusion-negative ALL patients (Figure 4B). Applying the same approach to the entire cohort, the AUCs for OS at 3, 5, and 10 years were 0.772, 0.816, and 0.887, respectively (Figure 4C). Patients were then classified into high-risk and low-risk groups based on the median risk score as the cutoff. A prognostic heatmap illustrated the correlation between risk scores and eight clinical characteristics in fusion-negative ALL, confirming that survival time decreased as risk scores increased (Figure 4D). Collectively, these results underscore the utility of CADPS mRNA levels in predictive modeling to more accurately assess clinical survival prognosis in FG-negative ALL, thereby supporting clinical decision-making with improved insight into survival outcomes.

### Function enrichment analysis

**Figure 5.**
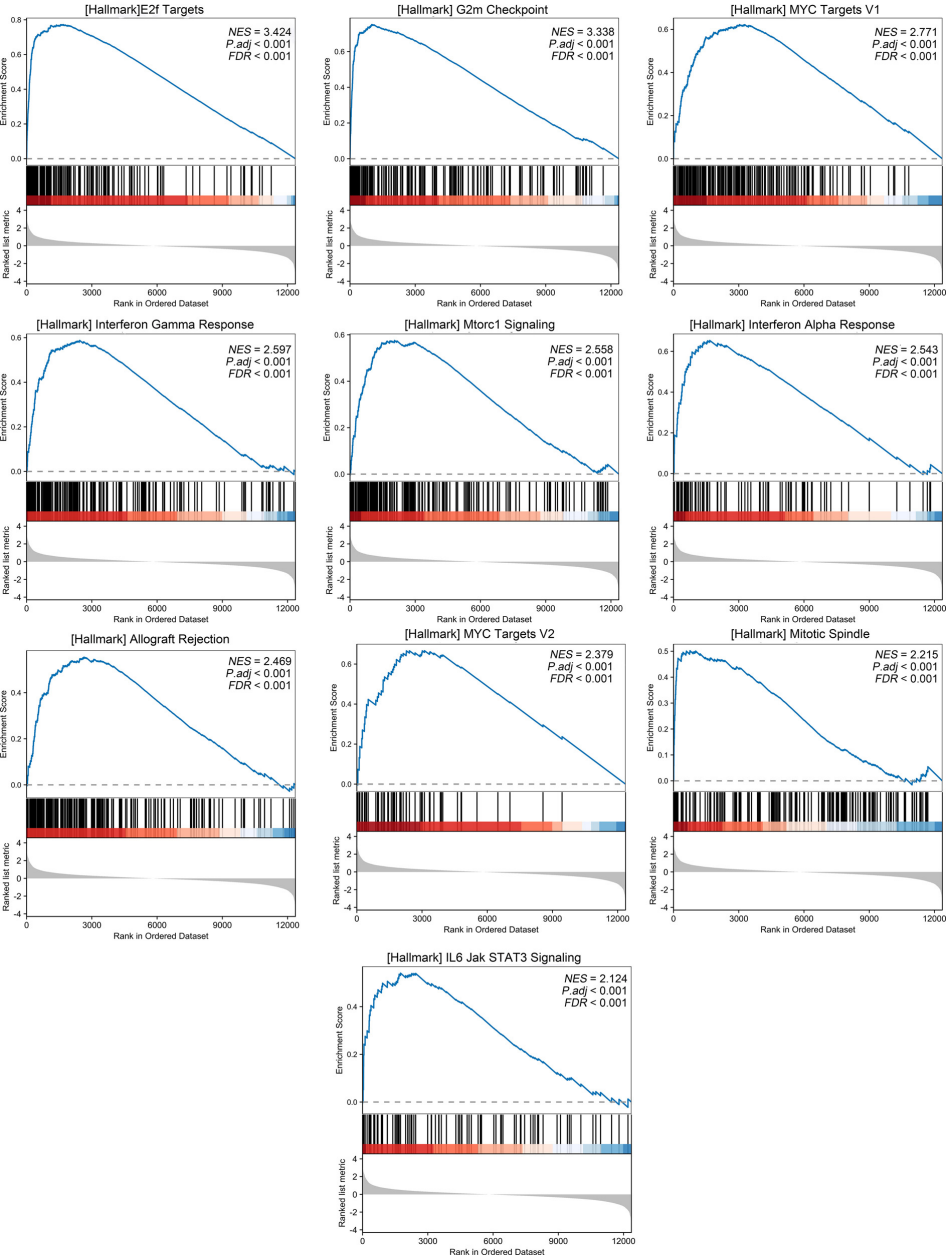
**Function enrichment analysis of low- and high-risk patients.** Differentially expressed genes between low- and high-risk pediatric ALL patients were analyzed using Gene Set Enrichment Analysis (GSEA) to identify hallmark pathways enriched in the high-risk group. The figure presents the top ten upregulated pathways, with enrichment plots illustrating the distribution of gene set members within the ranked gene list for each pathway. NES: Normalized enrichment score; FDR: False discovery rate.

To investigate the potential molecular mechanisms underlying the impact of the prognostic model on ALL progression, GSEA was performed on high- and low-risk groups based on the risk score. The top ten upregulated hallmark pathways showing significant differences between the two groups are presented in Figure 5. Notably, several biological functions related to proliferation and the cell cycle—such as E2F targets, mTORC1 signaling, IL6-JAK-STAT signaling, and G2/M checkpoint regulation—were significantly enriched in the high-risk group compared to the low-risk group.

**Table 3:** Univariate and multivariate Cox regression analysis in entire ALL cohort

**Characteristics**	**Number**	**Univariate analysis**		**Multivariate analysis**	
		**HR (95% CI)**	***P* value**	**HR (95% CI)**	***P* value**
Age	163				
1≤age<10	106	Reference		Reference	
age≥10	57	2.164 (1.442–3.249)	**<** 0.001	1.959 (1.269–3.024)	0.002
Sex	163				
Male	83	Reference		Reference	
Female	80	0.523 (0.346–0.790)	0.002	0.615 (0.403–0.939)	0.024
WBC counts (x 10^9^/L)	163				
<50	116	Reference			
≥50	47	0.874 (0.554–1.378)	0.561		
Bone marrow relapse	163				
No	29	Reference		Reference	
Yes	134	5.819 (2.360–14.347)	**<**0.001	7.048 (2.831–17.547)	**<** 0.001
CNS relapse	163				
No	145	Reference			
Yes	18	1.043 (0.570–1.910)	0.891		
CNS status	163				
CNS 1	132	Reference		Reference	
CNS 2	28	1.287 (0.771–2.148)	0.335	1.297 (0.761–2.212)	0.340
CNS 3	3	0.000 (0.000–Inf)	0.996	0.000 (0.000–Inf)	0.994
MRD day 29	163				
<0.01	100	Reference			
≥0.01	63	1.093 (0.728–1.641)	0.667		
CADPS expression	163				
High	82	Reference		Reference	
Low	81	2.754 (1.814–4.182)	**<**0.001	2.329 (1.493–3.632)	**<** 0.001

### Prediction of drug sensitivity

**Figure 6.**
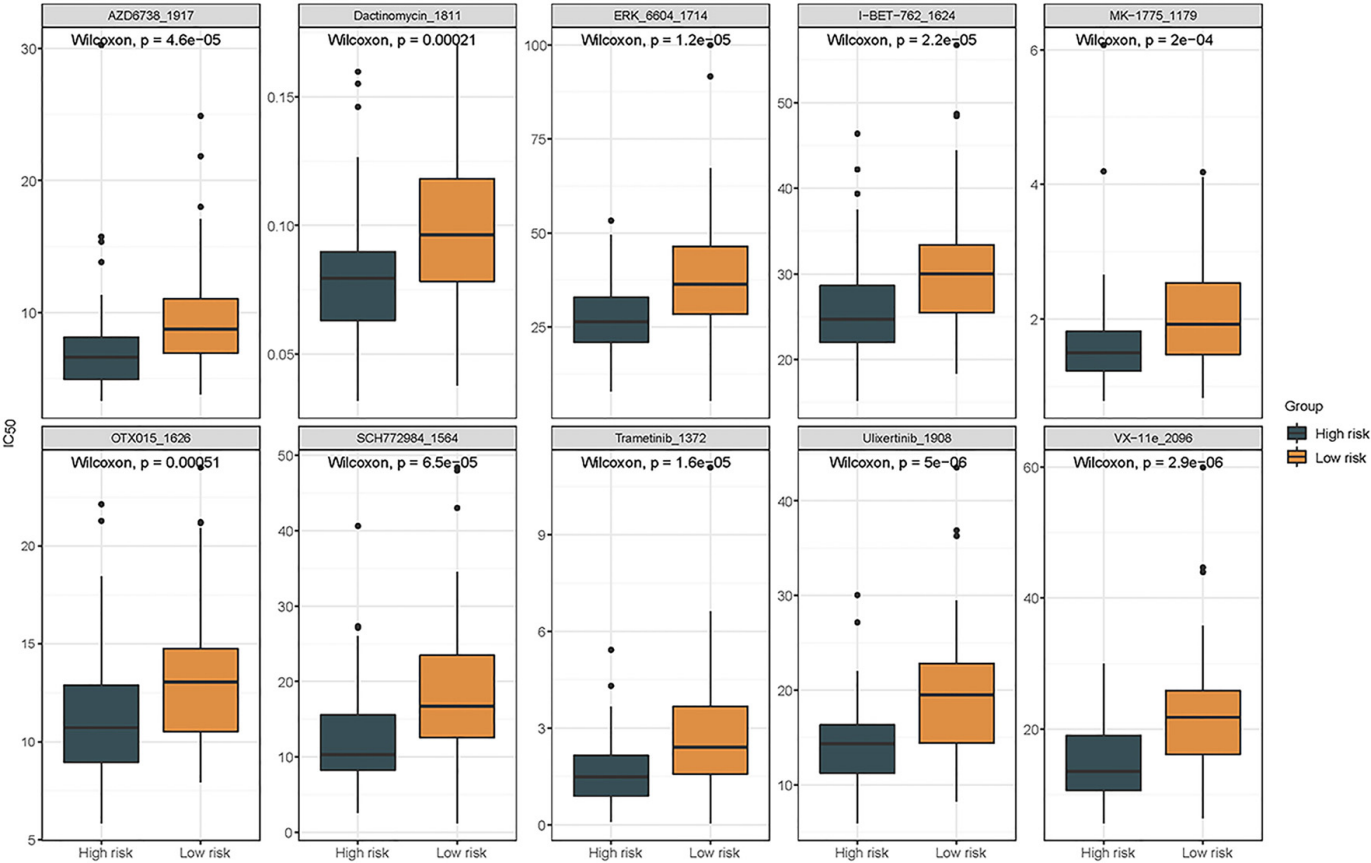
**The box plots displayed top 10 significantly different treatment-sensitive drugs between high- and low-risk groups.** Drug sensitivity (IC50) was inferred from transcriptomes using pRRophetic with GDSC references. The ten panels show the drugs with the clearest between-group differences; box plots depict per-patient predicted IC50 (median, IQR, whiskers; points ═ outliers) for high- vs low-risk groups. Comparisons were assessed by the Wilcoxon rank-sum test. IC50: Half-maximal inhibitory concentration; GDSC: Genomics of Drug Sensitivity in Cancer.

To assess the effectiveness of 198 chemotherapeutic and targeted-therapy drugs in pediatric ALL patients without FGs, the IC50 was calculated for each patient in the two groups based on transcriptome data (Table S3). Among the top 10 most significant drugs, patients in the high-risk group demonstrated greater sensitivity to AZD6738, Dactinomycin, and others compared to those in the low-risk group (Figure 6). These findings suggest that risk scores may be useful for predicting drug sensitivity in FG-negative ALL patients, with certain inhibitors potentially being more effective in the high-risk group.

**Figure 7.**
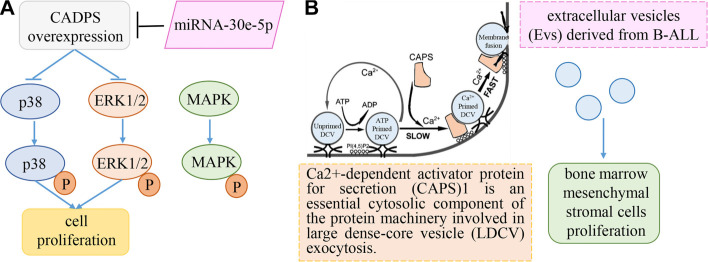
**Hypothetical mechanisms of CADPS in pediatric ALL.** (A) Cholangiocarcinoma-inspired model: CADPS overexpression suppresses tumorigenesis through the ERK/p38 MAPK signaling pathways. (B) Exosome-centric model: Based on the role of CADPS in vesicle secretion and the role of exosomes from B ALL in bone marrow mesenchymal cells.

## Discussion

While the mortality rate in children diagnosed with ALL is declining, accurately predicting survival outcomes remains a critical concern in clinical practice [19]. Numerous studies have sought to improve risk stratification by refining the classification of fusion subtypes and introducing new categorizations to better assess their prognostic implications [20–23]. A variety of FGs associated with pediatric ALL prognosis have been identified; however, it is noteworthy that approximately 40% of childhood ALL cases are FG-negative [6, 7]. In light of this, we conducted a study to investigate the relationship between genetic factors and relapse in FG-negative ALL patients.Our transcriptomic analysis revealed a significant downregulation of CADPS expression in recurrent cases of FG-negative ALL within our cohort. CADPS, also known as CAPS1, plays a critical role in the progression of various malignant tumors [16–18]. This EF-hand protein is involved in both Ca^2+^-phosphatidylinositol and cyclic adenosine 3′,5′-monophosphate (cAMP) signaling pathways, and is essential for regulating cellular proliferation and differentiation [24, 25]. Although no studies to date have shown that CADPS modulates chemoresistance or apoptotic pathways in leukemia cells, research in cholangiocarcinoma has demonstrated that CADPS is downregulated and associated with poor prognosis [17]. Moreover, its overexpression suppresses tumorigenesis through the ERK/p38 MAPK signaling pathways [17], suggesting a similar mechanism might be present in leukemia (Figure 7A). In addition, CADPS is a calcium-regulated protein that plays a key role in priming secretory vesicles, facilitating SNARE complex assembly, and mediating exocytosis via its Munc13-homology (MH) domain [26, 27]. Given these functions, we hypothesize that CADPS may also regulate the secretion of extracellular vesicles such as exosomes. Prior studies have shown that exosomes can enhance the proliferation and drug resistance of bone marrow mesenchymal stem cells through multiple signaling pathways, ultimately contributing to the high morbidity and mortality rates associated with ALL [28] (Figure 7B). Furthermore, our GSEA analysis indicated that the high-risk group was enriched for pathways related to proliferation and the cell cycle (Figure 5), further supporting a potential role for CADPS in leukemogenesis, possibly via exocytosis or ERK/p38 MAPK signaling. These proposed mechanisms, however, require experimental validation. We plan to initiate collaborative research in future studies to explore the role of CADPS in leukemogenesis and chemoresistance.

**Table 4:** Univariate and multivariate Cox regression analysis in FG-negative ALL cohort

**Characteristics**	**Number**	**Univariate analysis**		**Multivariate analysis**	
		**HR (95% CI)**	***P* value**	**HR (95% CI)**	***P* value**
Age					
1≤age<10	74	Reference		Reference	
age≥10	55	2.566 (1.617–4.073)	**<** 0.001	1.970 (1.194–3.250)	0.008
Gender					
Male	61	Reference		Reference	
Female	68	0.522 (0.329–0.828)	0.006	0.570 (0.356–0.913)	0.019
WBC counts (x 10^9^/L)					
<50	89	Reference			
≥50	40	0.855 (0.512–1.427)	0.548		
Bone marrow relapse					
No	27	Reference		Reference	
Yes	102	7.060 (2.573–19.377)	**<** 0.001	8.657 (3.136–23.901)	< 0.001
CNS relapse					
No	117	Reference			
Yes	12	1.506 (0.750–3.024)	0.250		
CNS status					
CNS 1	102	Reference			
CNS 2	25	1.362 (0.783–2.370)	0.274		
CNS 3	2	0.000 (0.000–Inf)	0.995		
MRD day 29					
<0.01	82	Reference			
≥0.01	47	1.034 (0.647–1.651)	0.890		
CADPS expression					
High	64	Reference		Reference	
Low	65	2.612 (1.625–4.197)	< 0.001	2.350 (1.406–3.930)	0.001

Validation against sequencing and clinical data from FG-negative childhood ALL cases in the TARGET database indicated that CADPS expression levels are an independent prognostic marker for pediatric ALL. We developed a prognostic prediction model using Cox regression analysis. ROC curve analysis demonstrated that the model effectively predicts patient outcomes, with significantly higher EFS and OS rates observed in children with elevated CADPS expression compared to those with lower levels.Although PCR-based assays or RNA panels could theoretically enable quantification of CADPS in clinical settings, routine implementation requires overcoming two major challenges: (1) establishing robust prognostic thresholds and (2) validating stable reference genes specific to fusion-negative ALL patients through systematic experimental studies. Future research will aim to standardize these parameters to help bridge the gap between biomarker discovery and clinical application. Chemotherapy remains the primary treatment for ALL; however, relapse driven by chemotherapy resistance is still the leading cause of mortality in this population [29]. To investigate potential mechanisms influencing prognosis, we stratified patients into low- and high-risk groups based on risk scores. GSEA revealed that DEGs were enriched in pathways related to cellular proliferation, including E2F targets, mTORC1 signaling, and the IL-6/JAK/STAT3 pathway—providing a possible explanation for the poorer outcomes observed in high-risk patients. Notably, the mTOR pathway is a key regulator of growth, proliferation, and apoptosis in B-precursor ALL cells [30]. Our analysis identified several top-ranked therapeutic candidates, including AZD6738, Dactinomycin, MK-1775, Trametinib, Ulixertinib, and VX-11e. Among them, Dactinomycin—widely used in pediatric solid tumors with an established safety profile—also demonstrates significant activity in infant ALL cell lines. In patient-derived xenograft models, administration of Dactinomycin at 18 µg/kg weekly or biweekly conferred modest but statistically significant survival benefits [31]. Preclinical studies have shown that the BRD4 inhibitor I-BET762 (GSK525762A) effectively eliminates common B-ALL cells and suppresses tumor cell proliferation [32]. Additionally, the WEE1 inhibitor MK-1775 has been found to induce sustained growth inhibition specifically in KMT2A-rearranged B-ALL [33]. Exposure to OTX015 has also resulted in growth inhibition, cell cycle arrest, and apoptosis at submicromolar concentrations in both acute leukemia cell lines and patient-derived leukemic samples [34, 35]. Trametinib, a selective MEK inhibitor, has demonstrated preclinical efficacy against RAS-mutant, MLL-rearranged ALL within specific microenvironmental niches, where it effectively reduces ERK phosphorylation *in vivo* [36, 37]. Furthermore, a notable case study reported successful treatment of Ph-positive B-ALL using a combination of trametinib and dasatinib alongside conventional chemotherapy [35].

AZD6738 (Ceralasertib), a selective inhibitor of ataxia telangiectasia and Rad3-related protein (ATR), has demonstrated synergistic preclinical activity with a Bruton tyrosine kinase (BTK) inhibitor in TP53- and ATM-defective CLL cells (NCT03328273). Ulixertinib (BVD-523), a potent and highly selective ERK1/2 inhibitor, was shown to inhibit ERK phosphorylation *in vitro* across all CLL cases harboring mutations in genes of the RAS-BRAF-MAPK-ERK pathway [38]. Although ERK_6604, SCH772984, and VX-11e currently lack studies in B-ALL, their potential efficacy merits further investigation. In summary, Dactinomycin, I-BET762, MK-1775, OTX015, and Trametinib may hold promise for the treatment of pediatric ALL and warrant deeper exploration in future studies. Our study has several limitations. First, the sample size of our single-center cohort is relatively modest—particularly the relapse subgroup (*n* ═ 10)—and no formal power calculation was performed due to the retrospective nature of the study. While this limitation was partially addressed through validation in the independent TARGET cohort (*n* ═ 129, FG-negative ALL), future prospective studies with larger cohorts are needed to increase statistical power. Second, the lack of long-term clinical follow-up limits our ability to evaluate recurrence and survival trends. Moreover, incomplete karyotype data and missing information on IKZF1 mutations in the TARGET database prevented us from assessing the clinical relevance of CADPS expression in relation to current risk stratification tools (e.g., hyperdiploidy or IKZF1 status). However, we did analyze survival outcomes in subgroups of patients from the TARGET dataset with trisomy of chromosomes 4 and 10, or with *ETV6/RUNX1* fusion—both associated with favorable prognosis. The results showed that CADPS expression could further stratify patients with trisomy 4 and 10, but not those with *ETV6/RUNX1* fusion. Finally, although our findings identify CADPS as a potential prognostic biomarker in FG-negative ALL, we did not perform functional experiments to confirm a causal role for CADPS in leukemogenesis or chemoresistance. Future studies should prioritize mechanistic validation—such as *in vitro* knockdown/overexpression experiments in B-ALL cell lines and patient-derived xenograft models—to determine whether CADPS loss directly contributes to therapeutic resistance or disease progression.

## Conclusion

In conclusion, our findings suggest that CADPS expression may serve as a significant prognostic marker for evaluating clinical outcomes in pediatric ALL patients without detectable FGs. To the best of our knowledge, this study is the first to investigate the implications of prognostic biomarkers in pediatric ALL patients lacking identifiable gene fusions.

## Supplemental data

Supplemental data is available on the following link: https://www.bjbms.org/ojs/index.php/bjbms/article/view/12254/3904

https://www.bjbms.org/ojs/index.php/bjbms/article/view/12254/3905

https://www.bjbms.org/ojs/index.php/bjbms/article/view/12254/3906

## Data Availability

The in-house next-generation sequencing data are not publicly available due to ethical and local legal restrictions.
